# Electrodeposition of Rhodium Nanowires Arrays and Their Morphology-Dependent Hydrogen Evolution Activity

**DOI:** 10.3390/nano7050103

**Published:** 2017-05-03

**Authors:** Liqiu Zhang, Lichun Liu, Hongdan Wang, Hongxia Shen, Qiong Cheng, Chao Yan, Sungho Park

**Affiliations:** 1 College of Biological, Chemical Sciences and Engineering, Jiaxing University, Jiaxing 314001, Zhejiang, China; zhangliqiu.520@163.com (L.Z.); lichun.liu@mail.zjxu.edu.cn (L.L.); 18258320795@163.com (H.W.); qcheng_102@163.com (Q.C.); 2 Department of Chemistry, Sungkyunkwan University, Suwon 440-746, Korea; 3 School of Material Science and Engineering, Jiangsu University of Science and Technology, Zhenjiang 212003, Jiangsu, China; chaoyan@just.edu.cn

**Keywords:** rhodium nanowires, electrodeposition, citrate, HER, defect

## Abstract

This work reports on the electrodeposition of rhodium (Rh) nanowires with a controlled surface morphology synthesized using an anodic aluminum oxide (AAO) template. Vertically aligned Rh nanowires with a smooth and coarse morphology were successfully deposited by adjusting the electrode potential and the concentration of precursor ions and by involving a complexing reagent in the electrolyte solution. Scanning electron microscopy (SEM) and transmission electron microscopy (TEM) analyses were used to follow the morphological evolution of Rh nanowires. As a heterogeneous electrocatalyst for hydrogen evolution reactions (HER), the coarse Rh nanowire array exhibited an enhanced catalytic performance respect to smooth ones due to the larger surface area to mass ratio and the higher density of catalytically active defects, as evidenced by voltammetric measurements and TEM. Results suggest that the morphology of metallic nanomaterials could be readily engineered by electrodeposition. The controlled electrodeposition offers great potential for the development of an effective synthesis tool for heterogeneous catalysts with a superior performance for wide applications.

## 1. Introduction

Rhodium (Rh) is a well-known metal belonging to the platinum group and exhibiting outstanding catalytic properties in many important applications [1,2,3]. Despite its high cost, Rh is still attractive because it has specific chemical properties which allow the catalyzation of specific chemical reactions [4,5,6,7,8]. Moreover, Rh has several superior properties like chemical inertness towards mineral acids, low electrical resistance for electrical device applications and reflective properties for ornamental use. Similar to other noble metal catalysts, the high cost of Rh restrains its practical usage. To improve the utilization of Rh, intensive research has been focused on increasing the ratio between the surface area and the mass. Up to now, the most promising solution to increase the surface area has been to prepare Rh structures at the nanoscale, which can expose more atoms to the application environment. Many approaches have been reported to synthesize Rh nanocrystals suspended in liquid solutions [9,10,11,12,13] and other 1D nanowires [14,15] and nanotubes [16,17]. Rh crystals at the nanoscale size theoretically can provide high surface area per mass unit, but surface area is unavoidably lost due to the stacking of nanocrystals when immobilized onto a solid substrate for heterogeneous catalysis applications. Meanwhile, the electrical contact is generally weak between Rh nanocrystals and the conductive substrate. By contrast, the direct growth of a noble metal nanomaterial on a solid conductive substrate is better for achieving high-performance applications because of the high surface area, absence of aggregation, improved electrolyte accessibility and excellent electrical contact between the substrate and Rh.

Template-assisted electrodeposition is a convenient and powerful way to prepare one-dimensional metal nanomaterial on a conductive substrate. Anodic aluminum oxide (AAO) membrane with highly ordered and high-density nanopores is the most commonly used hard template to synthesize vertically aligned one-dimensional nanostructures [18,19,20,21]. The insulating nature of alumina allows one to electrochemically deposit inorganic (e.g., metal and metal oxides) and organic (e.g., conducting polymer) materials into a nanoporous AAO template. By using the AAO template-assisted electrodeposition strategy, smooth and solid cylindrical nanowires have been typically obtained. However, our previous results show that cylindrical nanopores allow nanostructures to be synthesized with other forms, such as nanotubes [22,23,24], nanobelts [25] and nanosprings [26]. These special forms of nanostructures usually require well-defined electrodeposition conditions. Rh nanowires synthesized using AAO templates have been previously reported [27,28], but no research have evidenced the synthesis of Rh nanowires with a controlled morphology. Therefore, this work demonstrates the synthesis of Rh nanowires with a controlled morphology by employing AAO template-assisted electrodeposition. The morphology of Rh nanowires could be controlled by the concentration of electrolyte, the electrode potential and the use of a complexing reagent. A coarse Rh nanowire array exhibited superior performance as an electrocatalyst for the hydrogen evolution reaction, taking advantage of a greater surface-to-mass ratio and higher density of structural defects compared to smooth Rh nanowires. 

## 2. Results and Discussion

### 2.1. Influence of Citrate on the Morphology of Rh Nanowires

Citrate has been extensively used as a reducing and complexing reagent for the synthesis of noble metal nanoparticles [29,30]. It has a strong tendency to complex with metal cations and be adsorbed on metal surfaces, especially on the (111) facets of noble metals [31,32]. Inspired by the use of citrate as a capping reagent to influence specific facets of metal crystals, we attempted to introduce citrate into the electrolyte to promote the synthesis of anisotropic Rh nanowires using an AAO template-assisted electrodeposition. 

**Figure 1:**
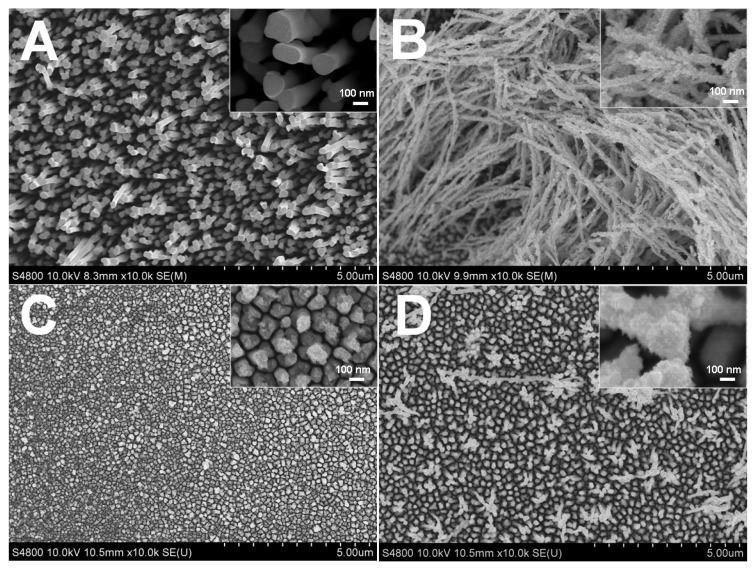
Scanning electron microscopy (SEM) images of rhodium (Rh) nanowires electrodeposited in aqueous electrolyte at −0.30 V for 2 C containing 1.6 mM RhCl_3_, 0.1 M HClO_4_, and different citrate concentration (**A**) 40 mM; (**B**) 16 mM; (**C**) 3.2 mM; (**D**) 0 mM.

Citrate in the electrolyte exerted a strong influence on the Rh electrodeposition kinetics and on the final morphology. The resulting nanostructures depended on the reduction rate of precursor ions [33]. Figure 1 shows the correlation between the morphology of Rh nanowires and the citrate concentration. The complexing degree between Rh(III) ions and citrate ions was enhanced when increasing the citrate concentration. The adsorption of Cl^−^ anions from the RhCl_3_ precursor on newly formed metal crystal during electrodeposition was limited, so Cl^−^ anion adsorption that usually leads to the formation of defects in the Rh nanostructures was suppressed, resulting in the formation of Rh nanowires with a smooth surface (Figure 1A). In contrast, Rh nanowires did not grow homogeneously and exhibited a distinct coarse morphology when citrate ions were absent or at a low concentration in the electrolyte (Figure 1B–D). This suggests that citrate has great influence on the growth of Rh nanowires. More specifically, when the citrate concentration was lower than 16 mM, Rh nanowires with irregular nanoscale branches and coarse surface were obtained (Figure 1B). In the same conditions, the diameter of resulting Rh nanowires was limited to <100 nm for an AAO nanopore diameter of about 200 nm. This is probably caused by the limited complexing ratio of citrate to Rh(III) at low concentrations, which allows Cl^−^ to participate in the complexing reaction to form citrate-Rh-Cl co-complexes. Under the influence of Cl^−^ and at low citrate concentration, Rh nanowires grew with a coarse surface. Moreover, the adsorption of citrate on specific facets of Rh crystals, such as (111), could possibly (fully or partially) block the access of Rh(III) adions to these facets. Such a blocking effect has been previously observed in noble metal nanoparticles synthesized by using an additive agent in the solution [31,32]. As a result, coarse and attenuated Rh nanowires were formed (Figure 1B). By further decreasing the citrate concentration to 3.2 mM, Rh nanowires were no better-aligned and a large area of the Au substrate was clearly not covered as shown in Figure 1C. This testifies that a moderate concentration (e.g., 16 mM) of citrate in the electrolyte is required to form coarse and well-aligned Rh nanowires at a potential −0.3 V. When citrate was absent in electrolyte, sparse Rh nanowires were produced using the same electrode potential (Figure 1D). 

**Figure 2:**
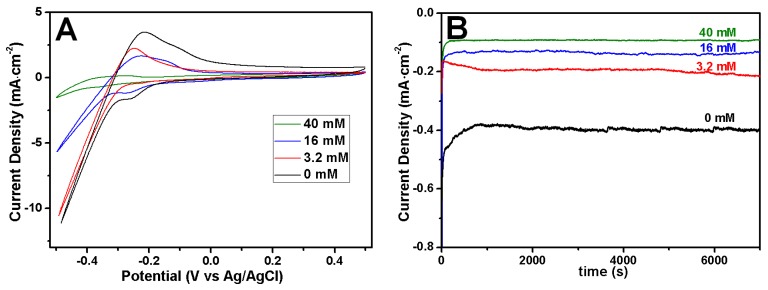
(**A**) Cyclic voltamogramms obtained using Au film-supported anodic aluminum oxide (AAO) template as a working electrode at different citrate concentrations; (**B**) Current profiles of AAO template-assisted electrodeposition using different citrate concentrations in the electrolyte, 40 mM, 16 mM, 3.2 mM and 0 mM. The concentration of RhCl_3_ and HClO_4_ was fixed at 1.6 mM and 0.1 M, and electrode potential was −0.3 V.

The morphology dependence of Rh nanowires from the citrate concentration was also closely related to the reduction rate of Rh ions. The Reduction rate of ions or the generation rate of atoms affects the morphology of Rh nanowires. Cyclic voltammetry (Figure 2A) and chronoamperometry (Figure 2B) were conducted to observe how citrate concentrations affect the reduction of Rh ions. The current level at −0.5 V decreased when increasing the citrate concentrations from 0 to 40 mM (Figure 2A). This indicates that the presence of citrate makes the Rh(III) ions reduction more difficult. At a given electrode potential and a high citrate concentration, more negative ionic Rh complexes were formed, which led to the decrease of the reduction rate (or the current density) (Figure 2B). At a low reduction rate, Rh atoms can have a sufficient time to choose energy-preferred sites to grow and not irregularly accumulate. At a low current density (green trace in Figure 2B) the formation of smooth Rh nanowire is facilitated as shown in Figure 1A. At high current density (blue curve in Figure 2B) well-formed coarse and thin Rh nanowires are obtained. At higher current density (red and black curves in Figure 2B) Rh nanowires deposition was unsuccessful. Since citrate coordination with Rh ions shifts the electrode potential to lower values, at a constant electrodeposition potential relative to reference electrode (Ag/AgCl) the current density is different from each control electrolyte. In order to keep an equivalent actual electrode potential for each citrate-containing electrolyte, we operated the electrodeposition in constant overpotential conditions (OCP). As a result, Rh nanowires deposited from different concentrations of citrate in electrolyte had a similarly smooth morphology.

### 2.2. Influence of Electrode Potential on Morphology of Rh Nanowires

**Figure 3:**
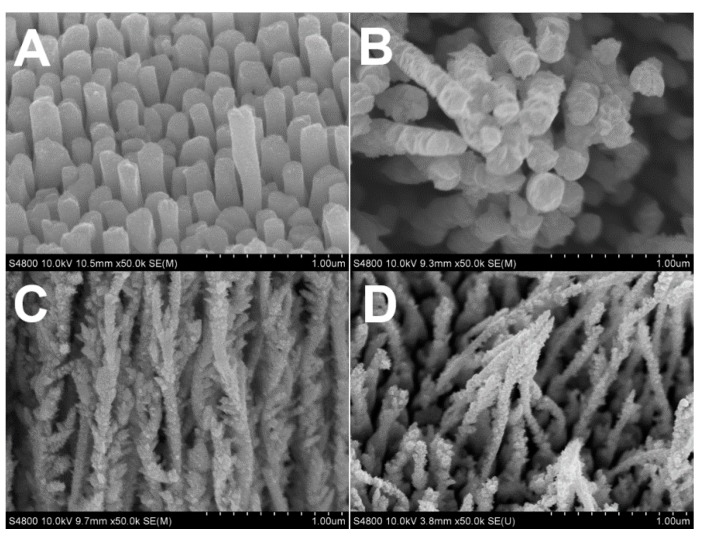
SEM images of Rh nanowires electrodeposited at different potentials in aqueous solution containing 1.6 mM RhCl_3_, 16 mM sodium citrate and 0.1 M HClO_4_. The constant growth potential: (**A**) −0.20 V; (**B**) −0.25 V; (**C**) −0.30 V; (**D**) −0.50 V.

The electrode potential is strongly correlated with the kinetics of Rh atom generation [33,34]. It is then necessary to evaluate the influence of the electrode potential on the Rh nanowires’ morphology. The influence of the electrode potential on the morphology of Rh nanowires was evaluated using the electrolyte containing 16 mM citrate that resulted in a well-defined coarse morphology. Figure 3 shows SEM images of Rh nanowires deposited at different electrode potentials in the presence of citrate. When the growth potential was set at −0.20 V, Rh nanowires had a regular shape and were relatively smooth (Figure 3A). When the potential was shifted by only 0.05 V to −0.25 V, Rh nanowires became much coarser (Figure 3B). This indicates that the growth of Rh nanowires is strongly affected by the electrode potential. This phenomenon was even more obvious when the growth potential was set at −0.30 V and −0.5 V, causing a strong branching effect as indicated in Figure 3C,D. This shows that when the deposition potential was more negative, the Rh(III) reduction was more rapid, which translates into a coarser morphology. When the deposition potential is more positive, the Rh(III) reduction is slowed, which allows the formation of smooth Rh nanowires. We conclude that the morphology of Rh nanowires depends not only on the citrate concentration, but also on electrode potential.

### 2.3. Concentration Effect of Rh(III)

**Figure 4:**
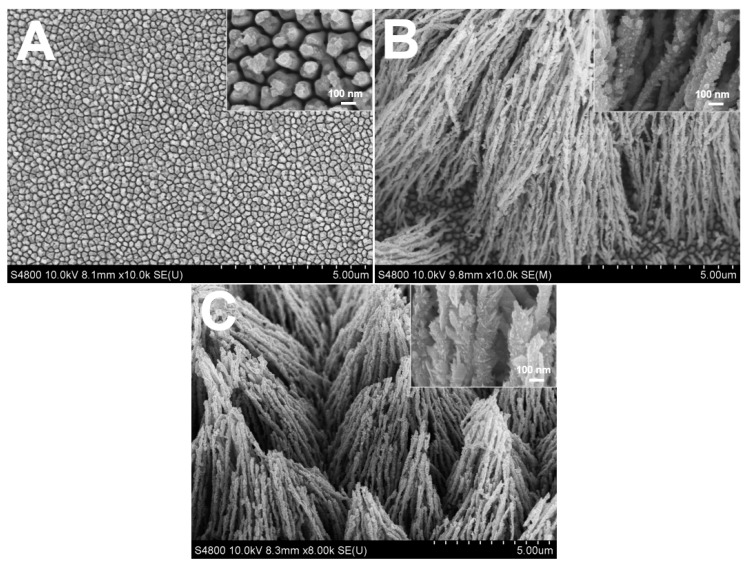
SEM images of Rh nanowires electrodeposited at −0.30 V for 2 C using a solution containing 0.1 M HClO_4_, 16 mM sodium citrate and different concentration of RhCl_3_. (**A**) 0.8 mM; (**B**) 1.6 mM; (**C**) 3.2 mM.

From the above results, the 16 mM sodium citrate electrolyte was used to evaluate how the concentration of Rh(III) affects the Rh nanowires’ morphology (Figure 4). At a low Rh(III) concentration of 0.8 mM, small Rh nanoparticles with irregular shapes were synthesized and Rh nanowires were not formed, similar to what was observed when citrate was absent from the electrolyte. Large areas of the Au substrate were clearly observed through the particles interspace (Figure 4A). This suggests that, at low Rh(III) concentration, the growth rate of Rh was too slow to effectively form nanowire structures. As the concentration of Rh(III) was increased to 1.6 mM, Rh nanowires with rice spike shape were mainly formed (Figure 4B). However, when increasing the concentration of Rh(III) to 3.2 mM, very loose Rh nanowires were formed and tended to collapse (Figure 4C). Above 3.2 mM, the morphology of Rh nanowires was similar to what is shown in Figure 4A. From these experimental results, the optimum conditions of 16 mM citrate concentration, 1.6 mM Rh(III) and −0.3 V potential were selected to produce well-shaped coarse Rh nanowires.

**Figure 5:**
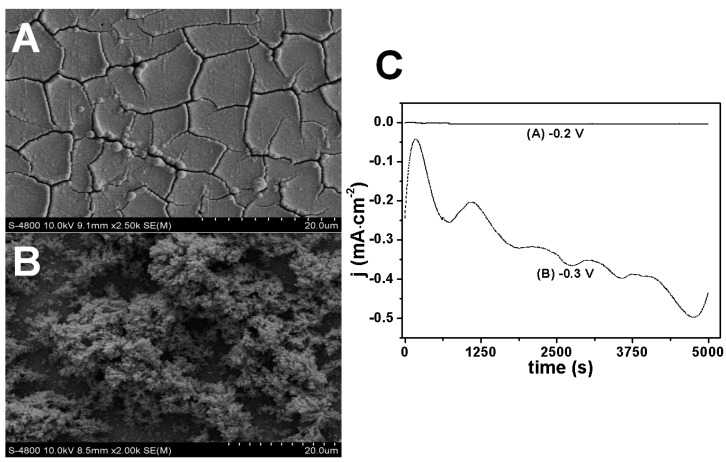
SEM images of Rh deposits on glassy carbon without assistance from the AAO template at different electrode potentials (**A**) −0.2 V; (**B**) −0.3 V, (**C**) shows current profiles during the electrodeposition. The electrolyte contains 1.6 mM of RhCl_3_, 16 mM of sodium citrate and 0.1 M of HClO_4_.

The coarse morphology of Rh nanowires was correlated to the electrolyte composition, the Rh(III) concentration and the electrodeposition potential. AAO nanopores played the role of a nanoscale container, which is not responsible for the formation of coarse or smooth morphologies. This was verified by electrodepositing Rh nanostructures on a planar glassy carbon electrode without the assistance of the AAO template (Figure 5). Figure 5A shows a relatively smooth surface at −0.2 V, while Figure 5B shows a porous bush-like Rh deposit at −0.3 V. This suggests that the morphology of Rh nanowires is strongly affected by electrodeposition condition, but not the presence of the AAO template. Apparently, coarse Rh deposits have a higher surface area, which contributes to the enhanced current level observed during the electrodeposition as shown in Figure 5C (dashed line). The current difference was also partially due to the potential difference. The high current led to a strong hydrogen evolution, and hydrogen bubbles influenced the stability of the current level, resulting in the wrinkled current curve in Figure 5C.

### 2.4. pH Influence on the Electrodeposition of Rh Nanowires

**Figure 6:**
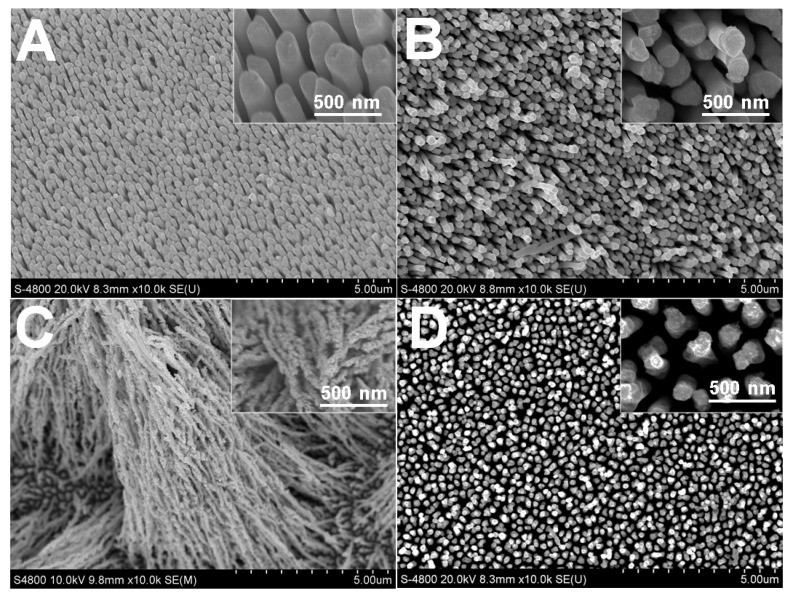
Morphological change of Rh nanowires electrochemically deposited at −0.3 V using electrolyte solution with a constant pH 1.37 and with different concentration of trisodium citrate, (**A**) 0 mM; (**B**) 3.2 mM; (**C**) 16 mM; (**D**) 40 mM.

**Figure 7:**
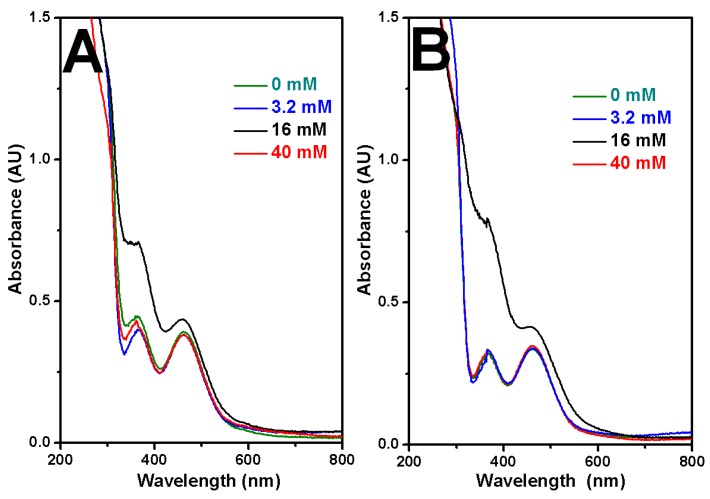
Ultraviolet-visible (UV-Vis) spectra of electrolytes without controlling pH (same as used in Figure 2) (**A**) and with constant pH 1.37 (same as used in Figure 6) (**B**).

Since pH is one of the most important parameters for an electrolyte, pH influence on the morphology of Rh nanowires was further investigated based on the optimal electrode potential and concentration of electrolytes established from above experiments. Addition of trisodium citrate into electrolytes caused increases in pH values. pH reached 1.12, 1.15, 1.37, 3.05 when 0, 3.2, 16, 40 mM trisodium citrate were in the electrolyte of 0.1 M HClO_4_ and 1.6 mM RhCl_3_. The morphologies of electrodeposited Rh nanowires were obtained, as shown in Figure 6, using constant pH 1.37 that was adjusted by adding NaOH or HClO_4_. At constant pH, only 16 mM citrate in the electrolyte could produce coarser Rh nanowires with attenuated diameter and many branches (Figure 6). This experimental set suggested that the concentration of trisodium citrate, pH of electrolyte and electrode potential of electrodeposition strongly correlate with the formation of coarse Rh nanowires. pH did influence the coordination between Rh(III) and ligands. Ultraviolet-visible (UV-Vis) spectrophotometry further evidenced that 16 mM citrate containing electrolyte is special and feasible to give a distinct morphology of Rh nanowires (Figure 7). The absorbance at 361 nm and 462 nm originates from d-d transition in RhCl_3_ compound. Without keeping pH constant, 16 mM citrate in the electrolyte gave rise to the strongest absorbance at 361 nm (black trace, Figure 7A). This unexpected result was probably caused by the formation of Rh(III)-citrate-Cl co-complex at the specific pH 1.37, which induced the strongest absorption of photons of light. Only a small absorbance difference was observed among other three electrolytes, but these absorbances significantly differ from what 16 mM citrate electrolyte gave. At a constant pH 1.37, the similar result was obtained as shown in Figure 7B. 16 mM citrate containing electrolyte still gave the strongest absorbance and other three control electrolytes showed tiny difference in absorbance. Conclusively, the combination of proper concentration of RhCl_3_, trisodium citrate, perchloric acid in the electrolyte, and proper pH, and proper electrode potential can synergistically produce coarse Rh nanowires utilizing AAO template-assisted electrodeposition. 

### 2.5. XRD Characterization for Rh Nanowires

**Figure 8:**
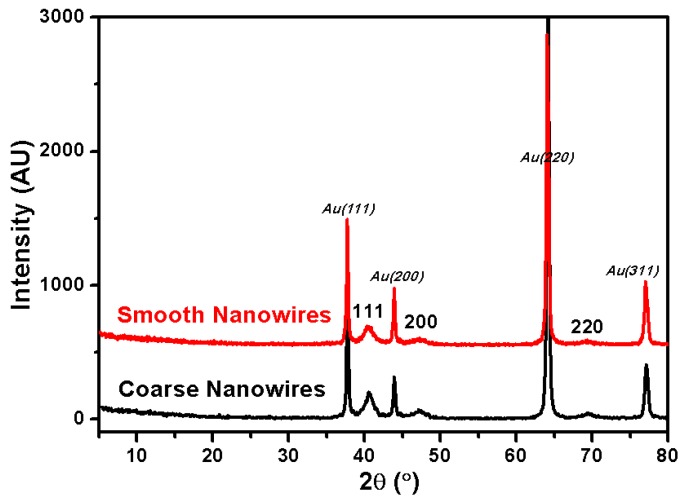
X-ray diffraction (XRD) patterns of Rh nanowires with smooth and coarse morphologies.

X-ray diffraction (XRD) patterns from smooth and coarse Rh nanowires were acquired to investigate their crystallinity (Figure 8). Four characteristic diffraction peaks from the metallic Au (2θ = 38.2°, 44.4°, 64.6° and 77.5°) were observed, corresponding to the Au (111), (200), (220), and (311) facets (JCPDS, No. 01-089-3697), respectively. In Figure 8, diffraction peaks at 2θ = 41.1°, 47.8°, 69.9° were ascribed to the Rh (111), (200) and (220) facets of the FCC Rh (JCPDS, No. 05-0685), respectively. Moreover, no other impurity peaks were detected, indicating the high phase purity of the deposit. Smooth and coarse Rh nanowires showed similar XRD patterns, indicating that the specific crystal facets couldn’t be screened in such electrodeposition condition. This is a difference from many metal nanoparticle synthesis methods where the citrate surfactant controls the nanoparticle shape.

### 2.6. TEM Characterization for Rh Nanowires

**Figure 9:**
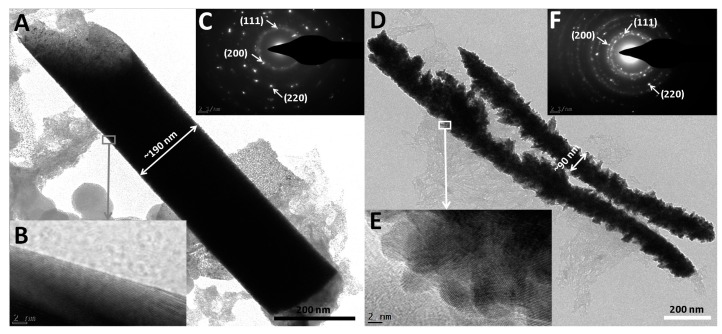
TEM images (**A**,**D**); high-resolution TEM images (**B**,**E**) and selected area electron diffraction (SAED) (**C**,**F**) for smooth Rh and coarse Rh nanowires, respectively.

We further carried out TEM characterization on smooth and coarse electrodeposited Rh nanowires. Figure 9A shows a smooth Rh nanowire with a diameter consistent with the nanopore size of the AAO template. On the other hand, coarse nanowires shown in Figure 9D had inconsistent and attenuated diameters and many branches were formed. The average diameter of coarse Rh nanowires (~90 nm) was much smaller than those of smooth ones (~190 nm). High resolution TEM images show that smooth Rh nanowires have a relatively longer range of ordered Rh atoms (Figure 9B). Coarse nanowires are obviously less crystallite (Figure 9E). Many electrodeposited metals were reported as polycrystalline in the literature. As-synthesized Rh nanowires were also polycrystalline, as observed by selected area electron diffraction (SAED) shown in Figure 9C,F.

### 2.7. Morphology-Dependent Hydrogen Evolution Activity

**Figure 10:**
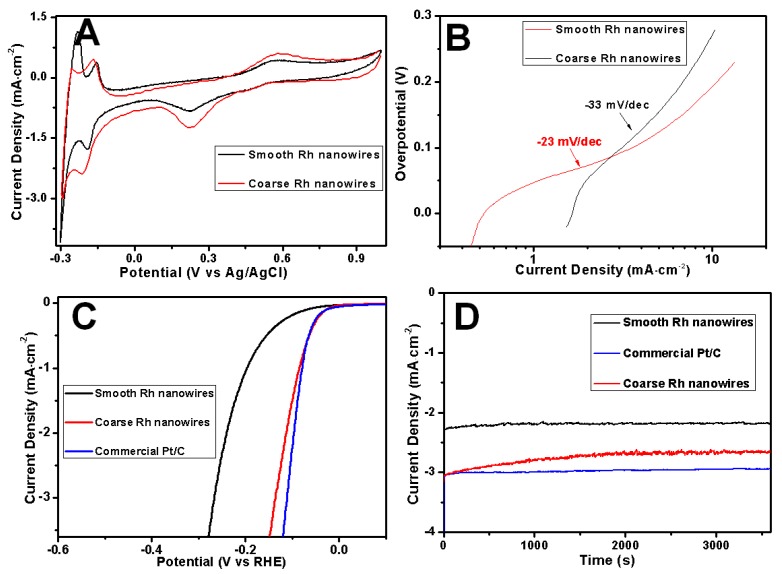
Cyclic voltammograms (CV) curves (**A**); Tafel curves (**B**); linear scanning voltammetry (LSV) curves (**C**,**D**) durability test of smooth (black) and coarse (red) Rh nanowires. The scanning was recorded at 2 mV·s^−1^ in 0.1 M H_2_SO_4_. Surface areas of the catalysts have been normalized.

Typical smooth (length: 1.1 (±0.2) μm, diameter: 223 (±39) nm) and coarse (length: 2.9 (±0.8) μm, diameter: 93 (±21) nm) Rh nanowires were used to investigate their hydrogen evolution activities depending on the morphology. Figure 10A shows the cyclic voltammograms (CV) from electrodeposited Rh nanowires with coarse and smooth surface measured in a 0.1 M H_2_SO_4_ aqueous solution at a scan rate of 2 mV/s. CV measurements show that the typical behavior of hydrogen adsorption/desorption and redox reactions occurring on Rh electrodes in an acidic electrolyte. Current peaks occurring between −0.3 V and 0 V are attributed to hydrogen adsorption (downward peaks) and desorption (upward peaks); the anodic peak at 0.58 V was ascribed to the oxidation of Rh and cathodic peak at 0.22 V was ascribed to the reduction of Rh oxide [28]. The CV curves for Rh nanowires electrodes were similar to that obtained with a Pt electrode. Hydrogen adsorption/desorption on Rh nanowires was associated with two peaks corresponding to strong and weak hydrogen molecules adsorption/desorption. In particular, the hydrogen binding energy (HBE) was found to be lower for coarse Rh nanowires as testified by the negative shift of the hydrogen desorption peak relatively to the one obtained in smooth Rh nanowires [35]. The decrease of HBE facilitates the hydrogen desorption from the catalyst surface. At the same surface area, smooth and coarse Rh nanowires weigh ~11.8 μg and ~4.1 μg, respectively. This implies that at the same mass level, the surface area of coarse Rh nanowires was substantially larger than smooth nanowires; this is due to differences in morphologies and in the average wire diameter. Also, hydrogen adsorption peak on coarse Rh nanowires is negatively shifted by 0.05 V, and the current level is higher than in smooth Rh nanowires. Such a peak shift is usually observed when a large amount of products is generated on an electrode. These facts indicate that coarse Rh nanowires outperform smooth ones on the catalytic performance versus HER.

Tafel slope is another key tool to investigate the intrinsic properties of an electrocatalyst by determining the rate-limiting steps of the HER [36]. Experimental Tafel slopes are displayed in Figure 9B. At a low overpotential, the Tafel slope obtained on coarse Rh nanowires electrode was as low as −23 mV/dec, while for the smooth Rh nanowires electrode it resulted −33 mV/dec. This obvious difference in Tafel slopes confirms that coarse Rh nanowires were more catalytically active versus the HER since a small Tafel slope is favorable in heterogeneous catalysis and leads to an enhanced HER rate at a moderately increased overpotential.

To further compare the performance difference versus the HER between smooth and coarse Rh nanowires, we carried out linear scanning voltammetry (LSV, Figure 10C). These measurements clearly show that coarse Rh nanowires (red curve) were more catalytically active than smooth ones (black curve). The onset potential of HER on coarse Rh nanowires resulted positively shifted (+0.14 V) respect to smooth nanowires. Furthermore, the HER catalytic activity of a commercial Pt/C composite was compared with the one obtained with Rh nanowires. Figure 10C indicated that coarse Rh nanowires had a similar catalytic activity to that of commercial Pt/C nanoparticles at a lower overpotential. Figure 10D presents the stability of hydrogen evolution on Rh nanowires at −0.2 V (vs. RHE). The current density on coarse Rh nanowires was ~22% higher than smooth Rh nanowires based on the same surface area level. This enhancement purely benefited from the higher catalytic activity of the coarse Rh nanowires. Owing to many nanoflakes and nanobranches existing on the coarse Rh nanowires, their current stability for hydrogen evolution was a bit worse than the smooth Rh nanowires and commercial Pt/C catalyst, as evidenced from the smoothness degree of current curve during the test period of time (3600 s) shown in Figure 10D. Since Pt is regarded as the best catalyst for HER [37], coarse Rh nanowires could be potentially applied in high-performance electrocatalysts for HER. In addition, structural defects in coarse Rh nanowires also partially contributed to enhanced catalytic activity versus HER [38]. The density of defects in smooth and coarse Rh nanowires is distinct, as compared between Figure 9B,E. In Figure 10, the consistence between LSV, CV, and chronoamperic measurements revealed that HER activity on Rh nanowires depended on their surface morphologies and coarse Rh nanowires exhibited a higher catalytic activity.

## 3. Materials and Methods 

### 3.1. Chemicals

RhCl_3_·3H_2_O (99.99%) was purchased from Alfa Aesar, Haverhill, MA, USA. Sodium citrate (C_6_H_5_Na_3_O_7_·2H_2_O), perchloric acid (HClO_4_) and all other chemicals were purchased from Sinopharm Chemical Reagent Beijing Co., Ltd., Beijing, China. Gold plating solution for Au predeposition was obtained from Technic, Inc. (Anaheim, CA, USA). All chemicals were of analytical reagent grade. The aqueous solutions were prepared with deionized (DI) water. The AAO (Whatman International Ltd., Maidstone, Kent, UK), with 60 μm thickness and ~200 nm pore diameter was used for all experimental trials.

### 3.2. Instruments

All electrochemical experiments were conducted on an electrochemical workstation P4000 (Princeton Applied Research, Bervyn, PA, USA) equipped with Pt mesh counter electrode and Ag/AgCl (3 M KCl) reference electrode. The morphologies of Rh nanowires were observed using Field Emission Scanning Electron Microscopy (FESEM, Hitachi S-4800, Chiyoda, Tokyo, Japan) operated at proper accelerating voltages. X-ray diffraction (XRD) analysis was performed using DX-2600 (Fangyuan, Dandong, China) X-ray diffractometer with Cu Kα radiation source. ICP-AES was IRIS Intrepid (Thermo Fisher, Waltham, MA, USA). The linear sweep voltammetry (LSV) for hydrogen evolution reaction (HER) was conducted with a Pine rotating disk electrode system (Pine Instruments Company, Grove City, PA, USA). High resolution TEM (HRTEM) and selected-area electron diffraction (SAED) were conducted on a JEOL-2100F transmission electron microscope (JEOL Ltd., Akishima, Tokyo, Japan) at an acceleration voltage of 200 kV. SHIMADZU 2550 UV-Vis spectrophotometer (SHIMADZU Corp., Nakagyo-ku, Kyoto, Japan) was used to collect UV-Vis spectra.

### 3.3. Methods

#### 3.3.1. Conducting Layer Fabrication

A stable aqueous solution of Au nanoparticles (d ~ 58 ± 11 nm) was prepared as follows. 10 mL of 20 mM HAuCl_4_∙3H_2_O solution was added to a 500 mL two-neck flask containing 415 mL of deionized water and a diamond-shaped magnetic stirring bar. 5 mL of 30.5 mM sodium citrate was added to the flask when the solution was boiling on a heating mantle while being vigorously stirred. After boiling for 10 min, the color changed to deep red, and the solution was then cooled to room temperature in an ice bath. The resulting ca. 58 nm Au nanoparticles were obtained in aqueous solution. 

Typically, ~10 mL of the above-mentioned Au nanoparticle solution was transferred into the stainless steel tube that contained AAO template for vacuum filtration. Under vacuum pressure, Au nanoparticles were blocked at the inlets of nanopores. Until all water from the Au solution in the tube was extracted into conical flask, the vacuum system was turned off and well-formed ~1 μm-thick Au nanoparticles conductive film was successfully formed on one side of the AAO template. There were not any nanoparticles entrapped in the nanopores of the AAO template. This method has been published elsewhere [39].

#### 3.3.2. Electrodeposition of Rh Nanowires

In a typical deposition procedure, Rh nanowires were synthesized by applying constant potentials using electrolyte containing 0.1 M HClO_4_, proper concentration of RhCl_3_ precursor and sodium citrate. The self-made Teflon electrochemical cell resembles that described by Liu et al. [40]. Graphitic plate as conductive lead and substrate was used to directly contact the Au nanoparticle-packed conductive layer and to support the AAO membrane in which Rh nanowires were deposited. The exposed geometric area of the working electrode was ~0.8 cm^2^. All electrodeposition was performed at room temperature (~20 °C) and in a standard, three-electrode configuration. Prior to the use of an AAO template, a conducting layer was prepared as described in 3.3.1. Then, Au predeposition (2 C·cm^−2^ at −0.95 V) using a commercial Au plating solution was conducted to fill the gaps among packed Au nanoparticles.

Rh nanowires were grown into pores of the AAO membrane by electrodeposition at a constant potential using an aqueous electrolyte containing Rh(III). After the nanowire deposition, the AAO templates were completely removed by immersing into 3 M NaOH solution for 15 min. The Rh nanowires was washed with deionized water for 5 times and dried at 50 °C in vacuum oven for further use.

#### 3.3.3. Characterization

All samples used for electrochemical measurements were without AAO support. The comparison between smooth and coarse Rh nanowires was conducted on the same electrical charge of the deposition. Different deposition conditions may produce varied amounts of net electrical charge for Rh deposition, so the normalization was performed by taking current efficiency into consideration.

The current efficiency (*CE*) was calculated using the equation shown below.

$$CE=\frac{m\cdot n\cdot F}{Q\cdot A}$$

where *m* is the deposited Rh metal mass (grams) determined by ICP technique through measuring Rh(III) concentration difference in the electrolyte before and after the electrodeposition, *Q* is the total charge passed through the circuit (coulombs), and *A* is the atomic weight of the metal element. *n* is the net amount of electrons transferred in the overall process per deposited metal atom, and *F* is the Faraday constant (96,485 C·mol^−1^).

#### 3.3.4. Electrode Fabrication for Dispersed Rh Nanowires

Pretreatment for a glassy carbon (GC) rotating electrode was completed sequentially by polishing using 50 nm alumina powder, deionized water rinse and sonication for 30 s, ethanol rinse and sonication for 30 s. Rh nanowires were released by etching out Au conducting layer using Au etching solution containing 4 mM I_2_ and 24 mM KI for 1 h. After five centrifugal cleanings, Rh nanowires were dispersed in 0.1 mL DI water. The 10 μL Rh nanowire-dispersed solution was dropped onto the GC electrode and evaporated water in a 60 °C oven. Then, Rh nanowires were fixed on the GC electrode by using 10 μL 0.5% Nafion ethanol solution.

#### 3.3.5 Surface Area Calculation Method

The electrochemical surface area (ECSA) was used to calibrate Rh nanowire surface area using a hydrogen monolayer adsorption/desorption method via the following equation [41]:

(1) $$ECSA=\frac{S_{ad}}{r\cdot c\cdot m}$$

where *S_ad_* denotes the integration area of a hydrogen desorption peak (expressed in units of V·A), *r* the scan rate used in cyclic votammetry (expressed in units of V·s^−1^), *c* the charge density (210 mC·cm^−2^) for hydrogen underpotential deposition region, and *m* the mass of working electrode material (g).

## 4. Conclusions

Rhodium nanowires with controlled morphology have been successfully synthesized by using anodic aluminum oxide template assisted electrodeposition. The electrode potential, concentration of the RhCl_3_ precursor and the concentration of sodium citrate (complexing agent) affected the morphology of Rh nanowires. At a moderate electrode potential and with a proper precursor concentration, favorable kinetics and growth rate of Rh atoms were obtained, allowing the formation of coarse Rh nanowires. Both an excessively high electrode potential and an excessively high citrate concentration led to the production of smooth Rh nanowires. The electrodeposition conditions have been optimized to obtain coarse-surface, rice-spike shape Rh nanowires. As-synthesized coarse Rh nanowires exhibited an enhanced catalytic performance toward hydrogen evolution reaction compared to smooth Rh nanowires. This was ascribed to the higher surface-to-mass ratio and to the higher density of active defect sites. This work shows that a controlled electrodeposition of metal nanowires is achievable and can provide a useful reference for the design of high-surface-area and defective nanomaterials.
